# Infodemiology of Influenza-like Illness: Utilizing Google Trends’ Big Data for Epidemic Surveillance

**DOI:** 10.3390/jcm13071946

**Published:** 2024-03-27

**Authors:** Dong-Her Shih, Yi-Huei Wu, Ting-Wei Wu, Shu-Chi Chang, Ming-Hung Shih

**Affiliations:** 1 Department of Information Management, National Yunlin University of Science and Technology, Douliu 64002, Taiwan; shihdh@yuntech.edu.tw (D.-H.S.); d11123001@yuntech.edu.tw (Y.-H.W.); m10623001@gemail.yuntech.edu.tw (S.-C.C.); 2 Department of Electrical and Computer Engineering, Iowa State University, 2520 Osborn Drive, Ames, IA 50011, USA; mshih@iastate.edu

**Keywords:** influenza-like illness, deep learning, infodemiology, Google Trends, ARIMA, big data

## Abstract

**Background:** Influenza-like illness (ILI) encompasses symptoms similar to influenza, affecting population health. Surveillance, including Google Trends (GT), offers insights into epidemic patterns. **Methods:** This study used multiple regression models to analyze the correlation between ILI incidents, GT keyword searches, and climate variables during influenza outbreaks. It compared the predictive capabilities of time-series and deep learning models against ILI emergency incidents. **Results:** The GT searches for “fever” and “cough” were significantly associated with ILI cases (*p* < 0.05). Temperature had a more substantial impact on ILI incidence than humidity. Among the tested models, ARIMA provided the best predictive power. **Conclusions:** GT and climate data can forecast ILI trends, aiding governmental decision making. Temperature is a crucial predictor, and ARIMA models excel in forecasting ILI incidences.

## 1. Introduction

The spread of and infection with infectious diseases threaten public health and economic stability in many countries [1]. Among infectious diseases, influenza is a significant global public health problem. According to estimates by the World Health Organization, many people around the world still suffer from influenza every year. The number of deaths ranges from 3 million to 5 million [2], and some studies have pointed out that accurately detecting the dynamics of seasonal and non-seasonal influenza outbreaks is still a considerable challenge [3,4,5]. In addition to the impact of influenza on human life and health, large-scale investment in preventive measures and medical resources also impacts the national economy [6], which is a considerable threat.

The terms influenza and influenza-like illness (ILI) are closely related, but distinct. While often used interchangeably, they denote different concepts. Influenza-like illness encompasses a broader category of symptoms, resembling those of the influenza virus but not exclusively caused by it [7]. ILI can be the result of various respiratory viruses besides influenza, such as the respiratory syncytial virus (RSV), rhinovirus, and adenovirus. The symptoms common to ILI are similar to those of flu and include fever, coughing, a sore throat, body aches, and fatigue, yet the underlying cause may not be influenza itself.

In the context of global health, monitoring ILI is crucial for maintaining health security worldwide. The early identification and management of ILI outbreaks is vital in curtailing the spread of respiratory viruses across borders and mitigating the threat of a worldwide pandemic. Given that ILI captures a spectrum of respiratory viruses beyond just influenza, surveillance efforts can distinguish between the various viral agents, such as influenza, RSV, and rhinovirus. This differentiation is essential for implementing precise public health interventions.

The surveillance of ILI is a key component of wider disease-monitoring initiatives, which aim to assess the incidence and spread of respiratory infections [8]. This surveillance supports health authorities in tracking infection trends, pinpointing populations at increased risk and efficiently distributing healthcare resources.

The widespread adoption of the internet has significantly enhanced convenience and access to knowledge-based services for its users worldwide. Individuals frequently utilize the “search” function of internet search engines to gather necessary information and derive knowledge from it. Consequently, over the past decade, the ease of access to health-related information online has transformed how individuals, public health professionals, and clinicians use the internet. Options for use include conducting online searches for personal health inquiries, understanding symptoms, and exploring related causes. Therefore, leveraging data from Google Trends (GT), a popular search engine, can offer valuable insights into the prevalence of medical conditions [9,10].

In recent years, more and more research has been conducted on using big data to analyze information in order to deal with the large amount of information currently available online. GT is a service based on big data that provides real-time trend predictions through the frequency of the ‘search keywords’ ability [11]. Nowadays, many research fields use real-time trends from GT data for analysis and prediction [1,5,12,13,14]. Due to the sensitivity and immediacy brought by search engine data, Eysenbach [15] pointed out that the data obtained by using Google search engine analysis can provide real-time and accurate predictions and reduce the cost of the entire flu season. The query data from the Google search engine are also widely used in research in fields such as economics and finance [16,17], being employed to monitor the incidence of influenza [1,2,18,19,20] and Dengue fever [6,12,21,22,23] and to forecast stock markets [24,25] and unemployment rates [26,27].

In the realm of influenza surveillance, various innovative approaches have been explored. Ghosh et al. [1] analyzed news articles using supervised temporal topic models and time-series regression in order to predict outbreaks. Yang et al. [2] and Ginsberg et al. [18] leveraged auto-regression and search data for real-time tracking and prediction, demonstrating the effectiveness of using internet-based data in early disease detection. Ortiz et al. [19] compared traditional CDC surveillance data with Google Flu Trends, while another study by Yang et al. [20] used Baidu search data and climate variables to obtain enhanced prediction capabilities. Integrating these diverse methodologies could significantly advance influenza surveillance efforts.

In preventing and controlling infectious diseases, it is essential to develop plans to control a disease before it becomes a pandemic. It takes 1 to 2 weeks for traditional surveillance data to be compiled into a database after an event occurs [28]. This delay limits a surveillance system’s ability to provide real-time information on the occurrence of infectious diseases, wasting a certain amount of time. Based on the resources and workforce of these units, the World Health Organization (WHO) estimates that, when an epidemic breaks out, these economic losses will be as high as USD 950 million. However, the issue worth paying attention to with influenza vaccines is that no one can say with absolute certainty which influenza viruses will circulate each year [29]. Therefore, even if people get vaccinated on time, there is still no guarantee that the vaccine will be effective during the epidemic season.

Previous studies have pointed out that there are potential errors in models constructed using only internet search data or climate factors [5,30,31,32,33]. In addition, data updates from traditional government monitoring stations are often delayed by one to two weeks [28], which may delay the availability of essential data. As in the 2009 and 2010 pandemic influenza outbreaks, the emergency rooms of major hospitals are experiencing shortages and congestion, which shows the importance of influenza prediction.

Infodemiology is the study of the distribution and determinants of information in an electronic medium, specifically the internet, with the aim of informing public health and public policy. This field analyzes web-based information to monitor and predict public health issues and trends. The infodemiology of influenza-like illness (ILI) refers to the study and analysis of information related to ILI that is generated and disseminated through digital platforms and sources, including search engine queries, social media posts, and online news [34]. This approach leverages big data and internet-based information to track, predict, and understand the patterns and dynamics of the ILI spread within populations. It aims to complement traditional epidemiological methods by providing real-time or near-real-time data, offering insights into public interest, concern, and behavior regarding ILI, and potentially enhancing disease surveillance and public health response strategies.

Among the available analytical methods, the deep learning LSTM model, as a type of recurrent neural network (RNN), is adept at recognizing and learning from sequential dependencies in data, making it particularly effective for time-series analysis. This capability renders LSTM models highly suitable for tracking and predicting phenomena like influenza outbreaks by analyzing historical patterns in search query data. LSTMs excel in modeling the intricate, nonlinear relationships that might exist between search trends and actual disease outbreaks, which simpler models might fail to accurately capture.

On the other hand, time-series analysis using ARIMA models excels in identifying and modeling the seasonal trends and cycles commonly observed in influenza outbreak data and related Google Trends search queries. ARIMA models are versatile and capable of handling time-series data with varying levels of trend and seasonality, thus offering valuable insights into the temporal dynamics of influenza-like illness outbreaks.

Multiple linear regression allows for the quantification of relationships between several independent variables (such as diverse GT keywords) and a dependent variable (like the number of ILI cases). This model is instrumental in pinpointing which keywords serve as significant predictors of outbreaks. In comparison to the LSTM and ARIMA models, multiple linear regression analysis is simpler and yields coefficients that are straightforward to interpret, facilitating an understanding of how each keyword impacts ILI outbreaks.

Each modeling approach brings unique strengths and has limitations. Employing these models in tandem allows for a comprehensive analysis, leveraging the strengths of one method to counterbalance the weaknesses of another. This synergistic approach enhances overall predictive capability and offers a more nuanced understanding of the interplay between public interest, as reflected in GT searches, and actual cases of influenza-like illnesses. The combined application of LSTM, ARIMA, and multiple linear regression models offers a holistic strategy for analyzing and forecasting influenza-like disease outbreaks, utilizing the distinct advantages of each method to achieve a deeper, more accurate interpretation of the data.

Hence, this study employs multiple regression models to examine the relationships among ILI emergency incidents, GT keyword searches, and climatic conditions during influenza-like outbreaks. It also assesses the predictive capabilities of these incidents through comparisons with the time-series ARIMA model and the deep learning LSTM model. The findings of this research could be shared with governmental health entities or medical professionals, helping to grasp the epidemic’s progression and informing subsequent decision making and planning.

## 2. Preliminary

### 2.1. Influenza and Influenza-like Illness

According to the World Health Organization (WHO), influenza is a viral disease that is fatal to specific groups of people. Every year, during an influenza outbreak, approximately 290,000 to 650,000 people worldwide die from respiratory-related illnesses. Another 3 million to 5 million people suffer from severe diseases, and 250,000 to 500,000 people die from them, accounting for about 5 to 10% of the patient cohort. These diseases range from mild to severe and can even cause death [35].

Influenza, commonly known as flu, is a viral infection that acutely infects the human respiratory tract (nose, throat, bronchi, and lungs). The infection is mainly caused by an infected person coughing or sneezing [36]. The incubation period for droplet infection is usually about 1 to 4 days, with an average of 2 days. The World Health Organization divides influenza into four types of seasonal influenza viruses (A, B, C, and D) according to their different pathogen classifications. “Influenza-like” refers to any virus that causes symptoms similar to those of influenza [37]. If an illness is as severe as influenza, it can be called influenza-like. The Taiwan Association of Family Medicine points out that influenza viruses cause more than 70% of influenza-like cases during influenza epidemics.

In addition, the rapid progression of influenza viruses often leads to sudden severe illness, which may lead to other concurrent diseases such as pneumonia, encephalitis, myocarditis, and ultimately death. Influenza is different from the common cold. Eccles [38] mentioned that different viruses and symptoms cause influenza. These viruses and their symptoms are more severe than the common cold and its effects, and their durations are also relatively long. The Agency for Disease Control and Prevention, which is under the Ministry of Health and Welfare, defines influenza as flu when fever (high fever for more than two days), pain (headache, noticeable muscle aches), and tiredness occur.

In temperate climates, seasonal influenza is mainly prevalent in winter, while sudden influenza may occur throughout the year in tropical regions. Previous studies have pointed out that influenza epidemics in Taiwan, Hong Kong, Singapore, and Japan occur almost every season [39]. Therefore, it is essential to establish a surveillance system for epidemic viruses. Since the emergence of the Spanish flu in 1918, the strain of the virus has been updated every year and has seen new developments, such as the Asian flu in 1958, the Hong Kong flu in 1968, the bird flu in 1997, and the H1N1 swine flu in 2009, all of which had severe outbreaks and caused more than one million deaths. Hsu et al. [40] also mentioned that the unpredictability and transmission potential of the next pandemic strain could result in a potential major natural disaster, and so the continuous and accurate surveillance of influenza viruses needs to be conducted.

The terms influenza-like illness (ILI) and influenza are related but not synonymous. They are often used interchangeably, but there are differences between them. Influenza-like illness (ILI) is a clinical term used to describe a group of symptoms commonly associated with influenza, including a range of symptoms that are similar to those caused by the influenza virus, but which are not necessarily caused by the influenza virus itself. ILI includes a spectrum of respiratory illnesses caused by viruses other than influenza, such as respiratory syncytial virus (RSV), rhinovirus, adenovirus, etc. The symptoms of ILI are often similar to those of the flu, including fever, cough, sore throat, body aches, and tiredness. However, the cause of ILI may not always be the flu.

The influenza virus is transmitted via coughing or sneezing, causing severe respiratory disease. The main periods of prevalence are the autumn and winter of each year. Getting the flu vaccine is a protective method and the first line of defense against the flu. However, even after receiving the flu vaccine, there is still a chance of contracting seasonal flu because the flu virus can be affected by various factors and mutate. The determinants of seasonal influenza remain complex, as the course of transmission is influenced by various factors, such as natural, social, and economic influences [41,42].

Previous studies, such as that of Zhang et al. [43], used time-series cross-correlation analysis and temporal risk analysis. This investigation of the relationship between search engines’ search index and climate variables, such as temperature and relative humidity, found a significant positive correlation between the outbreak of influenza and GT data. The search index and temperature data could be used to predict influenza events. Dai et al. [44] used the distributed hysteresis nonlinear model (DLNM) to explore the influence of ambient temperature on influenza and influenza-like illness (ILIs) cases in Jiangsu Province, China. They found that the increase in influenza-related activity in Jiangsu Province also led to an increase in ILI. In addition, influenza was more active when temperatures were lower; that is, influenza activity was negatively correlated with temperature, and other studies have used social media data (e.g., Twitter, GT influenza data), climate data (mean relative humidity, mean rainfall, etc.), and other data sources for the analysis and prediction of influenza correlations.

Many studies have also been based on different machine learning prediction methods in influenza transmission prediction. For example, Liu et al. [45] used wavelet analysis and linear detrending regression with the Fourier transform to predict the seasonal characteristics of influenza. Lu et al. [46] used the AutoRegression with General Online information (ARGO) method, combining influenza-related GT and electronic health records to predict influenza numbers in the United States. The results showed that combining influenza activity with temporal and spatial trends produced more predictions and reduced errors. The time-series method was also used to explore the effectiveness of the time-series regression technique in assessing the trend of time topics, and the results showed that cases can be predicted more accurately prior to the official report of the World Health Organization [1]. The relevant research on influenza epidemics and the methods used are summarized in Table 1.

In today’s interconnected global landscape, the tracking of influenza-like illness (ILI) plays a pivotal role in safeguarding public health. Prompt identification and intervention in response to ILI outbreaks are essential for curbing the cross-border transmission of respiratory viruses and minimizing the potential for a worldwide pandemic. ILI encompasses a variety of respiratory viruses beyond just influenza, including the respiratory syncytial virus (RSV), rhinovirus, and others. The ability to distinguish between these viral agents through ILI monitoring is crucial for deploying precise public health measures. Although the surveillance of influenza itself is vital, ILI surveillance extends the scope of disease-monitoring efforts. It aids in assessing the incidence and spread of respiratory infections, enabling health organizations to discern patterns, pinpoint populations at increased risk, and strategically distribute resources. By offering a comprehensive view of the respiratory disease burden, ILI surveillance facilitates a more agile and informed response from public health authorities to the ever-evolving challenge of respiratory virus transmission.

### 2.2. Google Trends and Its Applications

Google Trends (GT) was launched in 2006 to provide users with the function of searching information and data. Sullivan [55] pointed out that the annual search volume reached 1.2 trillion in 2012, and the search volume in 2016 reached 2 trillion. Nowadays, GT has become one of the popular resources for big data research. GT obtains information by analyzing the number of keyword searches. The immediacy of the information and the ease of operation of the interface attract many researchers. In addition to collecting a large amount of data, this method provides various sorting and comparison options [56]. Choi and Varian’s [57] study once again proved that internet search data are helpful in predictions. As for applying GT in disease prediction, Zhang et al. [43] found that temperature-related internet search indicators can predict influenza outbreaks and should be regarded as an effective indicator for modeling influenza outbreaks and monitoring and early warning systems; Ghosh et al. [1] also used relevant news trends to monitor the time-varying incidence of diseases. In addition to being used for medical research, GT can be used in other fields, such as explaining economic activities, energy use trends [11,58], agricultural economic development [14,59], and forecasting the unemployment rate [17,26,60,61], stock market changes [24,25,62,63], and tourism behavior [64,65,66,67].

### 2.3. Analytical Method

We use a combination of deep learning LSTM (long short-term memory) models, time-series analysis ARIMA (autoregressive comprehensive moving average) models, and multiple linear regression to identify associations between flu-like outbreaks and Google Trends (GT) keyword queries. Each approach has the advantage of providing a comprehensive understanding of the data. The following is a description of each analytical method.

#### 2.3.1. Multiple Regression Analysis

Multiple linear regression is a standard statistical analysis research method that uses mathematical expressions to understand the linear relationship between dependent and independent variables and study changes between variables. According to Jia et al. [68], regression analysis can extract meaningful information hidden in data to predict the dependent variable that will change with the independent variable. The general expression of multiple linear regression is shown in Equation (1):

(1) $${\hat{Y}}_{i}=a+b_{1}X_{1}+b_{2}X_{2}+b_{3}X_{3}+\ldots +b_{n}X_{n},\;i=1,\;\ldots ,n$$

where $b_{1},b_{2},b_{3}\ldots b_{n}$ are regression coefficients, indicating the predictive ability of $X_{1},X_{2},X_{3}\ldots X_{n}$ *n* variables on y.

#### 2.3.2. ARIMA (Autoregressive Integrated Moving Average Model)

**Figure 1:**
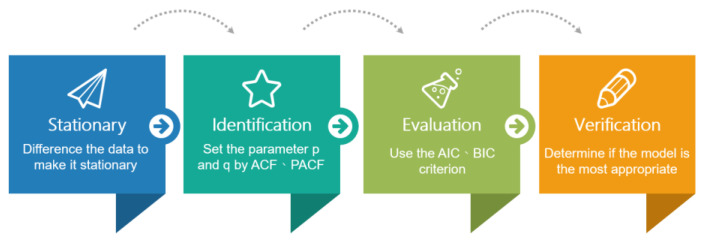
ARIMA model building process.

The ARIMA model was proposed by BOX and Jenkins in the early 1970s [69]. The ARIMA model forms a sequence of data using changing samples over time and then builds a regression model based on random error values and lag values in the data. The established model predicts the future value of the time series based on its past and present values [43]. ARIMA is composed of autoregressive (AR) and moving averages (MA), which contain three parts (p, d, q). These, respectively, represent the parameters of the autoregressive model, the order of the difference, and the moving average (moving average) parameters of the model. The process of establishing the ARIMA moving average autoregressive model is shown in Figure 1.

The ARIMA model is shown in Equation (2):

(2) $$\left(1-\sum_{i=1}^{p}\phi_{i}L^{i}\right){\left(1-L\right)}^{d}X_{t}=\left(1+\sum_{i=1}^{q}\theta_{i}L^{i}\right)\varepsilon_{t}$$

where L is the lag operator. The modeling steps are described as follows below.

1. Data stability processing

At this stage, the unit root test is used to determine the stationarity of the data. If the data appear unstable, differential processing is performed here, which is the d parameter in the ARIMA model. After differential processing, an ARIMA (p, q) model is generated. Difference indicates finding the difference between x(t) and x(t − 1).

2. Model identification

At this stage, AR (p) and MA (q) parameters are selected. After differential d processing is used to stabilize the data, stationary model data are obtained. Next, the autocorrelation function (ACF) and the local autocorrelation function (PACF) are used at this stage to determine the selection of coefficients and orders of AR (p) and MA (q).

The autocorrelation function (ACF), also known as serial correlation, is the correlation between a series of numbers n time intervals apart when delayed by n. The local autocorrelation function is obtained when the delay is n. In addition to considering the properties of the autocorrelation function, the values between intervals are also considered. The value is between 1 and −1, and the correlation coefficient is the basis for the strength of the association. The larger the absolute value is, the stronger the relationship between the two will be.

AR (p) represents the lag value (lag), which is the relationship between the current and historical values. Let us suppose that the time calculation is expressed in days. In that case, p = 1 represents the comparison between today’s data and yesterday’s data, and p = 2 represents today’s data compared with the data obtained from the day before yesterday, etc. MA (q) represents the accumulation of error terms in the autoregressive model, and the purpose of q is to eliminate random fluctuations in predictions. The selected p and q values are inserted into the model to obtain the results. The adjustment of the model’s parameters is performed in the next step.

3. Model evaluation

At this stage, the AIC (Akaike information criterion), AICc, or BIC method is generally used to identify suitable models. The smaller the value is, the more suitable the model will be for the data. Generally, the AIC criterion can evaluate the model’s results closer to the actual value. Then, the BIC criterion is used to find the best fit because it has more severe specifications in terms of parameter penalties.

4. Model validation

The last step is to verify the model’s results to determine whether the model is the most appropriate and select the best model for subsequent predictions.

#### 2.3.3. LSTM

**Figure 2:**
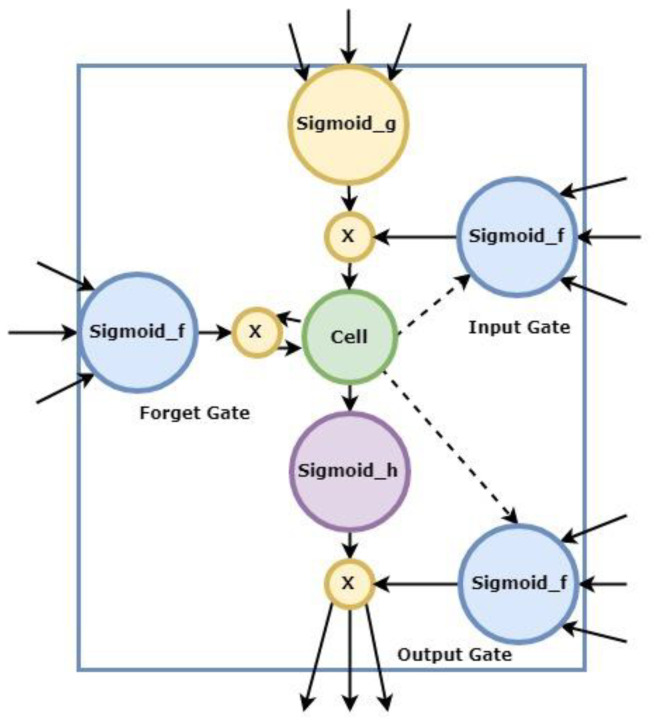
LSTM structure.

Long short-term memory (LSTM) networks are considered to be representative of deep learning, especially in sequential data processing, because of their ability to solve challenges related to vanishing gradients and capture long-term dependencies [70]. The long-term and short-term memory network technique is a learning sequence method composed of storage units. It strengthens decision-making effects by receiving information from the inside and memorizing it. The storage unit has three control valves (gates) for updating information and determining the memory. For storage and use, the control valves are the input gate, forget gate, and output gate. The long and short-term memory network unit structure is shown in Figure 2 below.

1. Input gate

The function of the input gate is to determine the importance of input information in relation to the entire block. Among the input “words”, the input “word” may or may not be necessary to the current data, and this judgment action is performed by the input gate (input gate) at this stage. This stage conducts preliminary data verification, identifies the importance of the information entered into the entire dataset, and decides whether to enter the data and generate memories.

2. Forget gate

The task of the forget gate is to identify whether the message that entered the memory program in the previous stage is a new topic or an opposite and unrelated word to the previous message. If the message meets the conditions above, it will be, at this stage, filtered and cleared. Otherwise, if it is judged to be new content, it will be retained in the memory.

3. Output gate

This stage controls which states in the internal memory will be transmitted to the output end and determines which of the messages stored in the memory unit will be output. The LSTM long short-term memory network model is generally expressed as Equations (3)–(8):

(3) $$f_{t}=\sigma \left(w_{f}\cdot \left[h_{t-1},x_{t}\right]+b_{f}\right)$$

(4) $$i_{t}=\sigma \left(W_{i}\cdot \left[h_{t-1},x_{t}\right]+b_{i}\right)$$

(5) $${\bar{C}}_{t}=tanh\left(W_{c}\cdot \left[h_{t-1},x_{t}\right]+b_{C}\right)$$

(6) $$C_{t}=f_{t}\times C_{t-1}+i_{t}\times {\bar{C}}_{t}$$

(7) $$o_{t}=\sigma \left(W_{o}\cdot \left[h_{t-1},x_{t}\right]+b_{o}\right)$$

(8) $$h_{t}=o_{t}\times \tan h\left(C_{t}\right)$$

In the input gate step process, Equation (3) identifies the input variables in order to determine whether the data should be input and remembered. In Equations (4) and (5), it is necessary to enter the forgetting gate to determine the new information to be created and save it in memory. In Equation (6), the memory information in the unit layer is updated. Equation (7), the information in the layer is used, and the output is prepared. In the final equation, Equation (8), the cell uses the tanh activation function to generate a value between 1 and −1. The above program, one part after another, is the internal structure of the LSTM method. Through the control state between gates, it remembers the information that needs to be remembered in a long-term sequence and forgets unimportant information.

LSTM has been widely used to predict events with time series and can be successfully applied. It also outperforms most non-parametric methods [71,72,73,74,75,76,77,78,79,80,81,82,83,84,85,86]. Related research, including flow forecasts, climate forecasts, and health monitoring, are shown in Table 2.

## 3. Materials and Methods

The utilization of LSTM, ARIMA, and multiple linear regression models presents a holistic strategy for analyzing and forecasting influenza-like disease outbreaks through Google Trends (GT) keyword searches. By harnessing the distinctive strengths of each model, this approach provides a nuanced understanding of the underlying data. The objective of this research is to employ predictive technologies in order to forecast the incidence of influenza-like illnesses. To achieve this, this study compares various predictive models, including the deep learning-based LSTM model and the ARIMA model for time-series analysis. Additionally, multiple linear regression is applied to the exploration of correlations between GT-related variables and the occurrence of influenza-like diseases.

### 3.1. Research Scenario

This study’s methodology unfolds through several key phases, beginning with data acquisition and preprocessing. Initially, we gather data on Google search trends, leveraging the number of searches for specific keywords within a given region on a particular day as our assessment metric. These data, along with governmental open data and climate information sourced from open platforms, undergoes preprocessing to ensure quality and relevance. Subsequently, the refined data are stored in a designated database, marking the transition to the phase of extensive data training and model construction.

During the data analytical phase, we employ a trio of research techniques to dissect and predict influenza trends: a deep learning long short-term memory (LSTM) network, a time-series analysis model using ARIMA, and multiple linear regression. These methodologies are chosen for their complementary strengths in capturing and forecasting the dynamic nature of influenza outbreaks based on the collected data.

**Figure 3:**
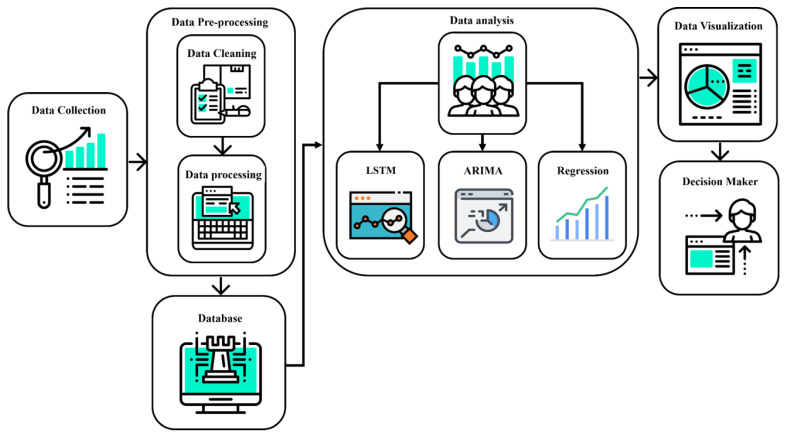
Research scenario.

The findings from this analysis are then visualized to facilitate understanding and are communicated to policymakers and health officials. This information serves as a valuable tool for informing public health strategies and policy implementation. The research scenario is encapsulated in Figure 3, illustrating the study’s comprehensive approach to the prediction of ILI epidemics.

### 3.2. Research Architecture and Modules

**Figure 4:**
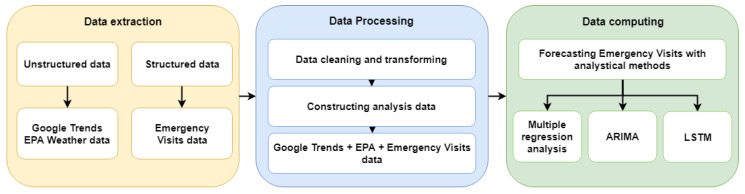
Research architecture.

This study’s research framework, depicted in Figure 4, outlines a comprehensive approach to analyzing ILI trends using open-source data. Initially, the process involves gathering GT data on keywords related to influenza-like symptoms from an open-source platform. These data undergo a preprocessing phase to ensure their effective storage in a database. The data collection is bifurcated into structured and unstructured categories. Structured data are sourced from the government’s open data platform, DATA.GOV.TW, which focuses on emergency department cases of ILI. Meanwhile, unstructured data encompass Google Trends keyword information on ILI trends and climate data from the Environmental Protection Agency of the Executive Yuan.

This study utilizes GT data to pinpoint the most pertinent search terms for ILI by conducting a keyword ratio analysis. Climate data are gathered from the Environmental Protection Agency’s global climate stations, with daily climate records being aggregated into weekly figures for analysis. These two distinct datasets are then merged and standardized through a data integration process. Subsequently, an optimal set of variables is selected to identify the key search indicators for tracking ILI trends.

The final phase of the study involves constructing predictive models and conducting predictive analysis. This is achieved by employing a multifaceted analytical approach that includes a multiple linear regression model, time-series analysis using an ARIMA model, and a LSTM network. These methods are applied to forecast the incidence of ILI, leveraging the integrated datasets for enhanced predictive accuracy.

### 3.3. Framework

**Figure 5:**
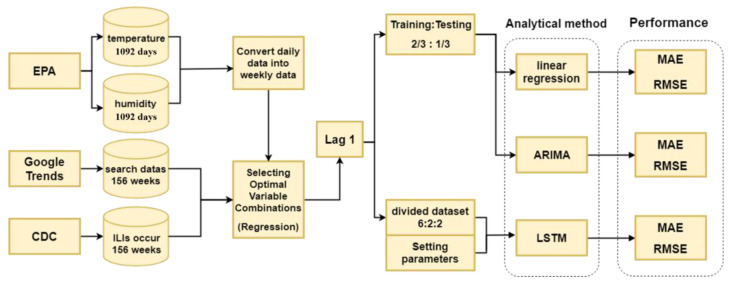
Research framework.

The framework used for the model used in this study is illustrated in Figure 5, which details a structured approach to forecasting influenza-like illness trends. The initial step in this involves aggregating data from three primary sources: keyword trends from the GT platform; the count of influenza-like illness cases from official health statistics; and environmental climate data. The first phase of the model’s framework focuses on data preprocessing. This includes addressing missing values, transforming daily climate data into weekly aggregates, and subsequently storing these processed data within a dedicated database. The aim is to prepare the raw data for use in further analysis by ensuring consistency and completeness.

In the second phase, the focus shifts to optimizing the selection of variables for the study. This involves exploring every possible combination of keywords and climate data variables to identify the set that offers the highest explanatory power. The chosen combination of variables then serves as the input for the models used in the subsequent analytical processes. The third phase entails determining the most effective lag time for use in predictions. This involves identifying the optimal time delay between observed data points and predictions to improve the model’s accuracy in forecasting future trends.

The fourth and final phase involves dividing the original dataset into two parts: one for training and the other for testing the models. The performance of three distinct analytical models is then assessed using common evaluation metrics such as the root-mean-square error (RMSE) and mean absolute error (MAE). This stage is crucial for evaluating the predictive accuracy and reliability of the models in terms of forecasting the incidence of influenza-like illnesses based on the selected variables and data preprocessing techniques.

## 4. Experimental Design

### 4.1. Dataset

The study sources its data from three primary locations: (1) Google Trends (GT), which provides data on trends related to influenza keywords from an open-source platform. Google Trends is a free tool developed and maintained by Google to show trends in the volume of searches for specific keywords or topics over time. It operates based on big data statistics from the Google search engine and provides trend data based on the volume of search queries. (2) Data on cases of influenza-like illnesses, which are made available by the Center for Disease Control (CDC) under the Ministry of Health and Welfare of Taiwan, are also used. The Taiwanese CDC’s infectious disease statistical information query system provides the general public, academia, the medical service industry, and public health departments with the most straightforward online query method with which to obtain the latest statistical information needed on Taiwan’s notifiable infectious disease. (3) Climate data from the Environmental Protection Agency of the Executive Yuan, which are accessible through its official website, are also used. Climatic data are used by the National Health Service and the Department of Disease Control to enhance the provision of weather forecasts and meteorological information. In light of the COVID-19 pandemic’s impact, which started in 2019, this study deliberately selects data from the years 2016 to 2018 in order to prevent any potential skewing of the analysis due to the pandemic’s influence. This period encompasses a total of 156 weeks of data. The specifics of the data’s sources, along with a detailed description of the variables used, are systematically outlined in Table 3.

#### 4.1.1. Emergency Influenza-like Cases

The Government Information Open Platform serves as an accessible, open-source repository, offering a wide array of Taiwanese statistical information to the public, academic circles, the healthcare sector, and public health authorities. This platform is characterized by its openness, providing data that are readily available and transparent. It facilitates the use of data by anyone with an interest or need, supporting modifications, sharing, and open access to encourage information exchange across various entities. This approach not only makes accessing information convenient but also contributes to enhancing quality of life. For this study, data were gathered on influenza-like illness emergency cases across all regions of Taiwan from the years 2016 to 2018. The compilation of this emergency case dataset drew upon survey data from the Centers for Disease Control (CDC), the Department of Health and Welfare, and the Agency for Disease Control. The dataset encompassed details such as the year and week of onset for the influenza-like illnesses, which were presented as string data, and the count of emergency visits attributed to influenza-like illnesses, which was recorded as numerical data.

#### 4.1.2. GT Keywords

GT captures trends through the search terms that users input, reflecting the frequency of these keywords within specific regions, on particular days, months, or over selected periods. The ratios of these keywords provide insights into fluctuating use trends over time, serving as a valuable predictive tool and reference point. This research focuses on the search volume of keywords across Taiwan from 2016 to 2018, with the GT data being sourced directly from Google. Following the methodology of Ginsberg et al. [16], cold and flu were initially identified as the primary search indicators. However, this study expanded its scope to include a broader range of influenza-like symptoms as defined by the CDC, such as fever, cough, muscle aches, headache, and fatigue. These symptoms are subsequently used as GT keyword search indicators, with a detailed exploration provided in the following section.

#### 4.1.3. Climate Variables

The Taiwan Ministry of Environment’s Environmental Information Open Platform (https://data.moenv.gov.tw/en, accessed on 31 January 2023) operates as a governmental open database, making a wide range of environmental information accessible to the general public, academic researchers, the medical community, and public health agencies. The platform features an array of integrated databases, categorized by environmental aspects (such as atmosphere, water, and land) or thematic areas (including pollution prevention and control, environmental statistics, ecological information, and monitoring activities). Users can search through and utilize this information according to their specific requirements.

For this study, meteorological data were gathered from 36 climate monitoring stations across Taiwan for the period from 2016 to 2018. The collected data included various parameters such as the monitoring station name, date of monitoring, station pressure, temperature, relative humidity, wind speed, precipitation levels, and hours of sunshine. This information was all derived from the Central Meteorological Administration’s survey data. The dataset incorporates details like the date and time of monitoring (treated as string data), along with temperature and relative humidity (considered numerical data), among others. Descriptive statistics for all variables collected within this timeframe are meticulously compiled in Table 4. This table includes a symptom’s variables, their minimum value (Min.), median (Median), average (Mean), maximum value (Max), variation (Var.), and standard deviation (SD).

### 4.2. Experimental Environment

This study’s experimental setup operates within a Windows 10 operating system environment, utilizing an Intel i5-3230 processor with a 2.6 GHz clock speed. For the training, testing, and prediction phases of the LSTM model, the open-source Integrated Development Environment (IDE) Spyder is employed to conduct experiments, with programming carried out in Python. Additionally, ARIMA models and multiple regression analyses are conducted using the IBM SPSS Statistics 22 software.

The experimental process begins with the construction of an initial prediction model, following the collection and preprocessing of data. This involves gathering relevant data in order to predict influenza-like illnesses, including official data on influenza-like emergency cases, GT keywords, and climate data, among other things. After the data preprocessing is completed, the experiment proceeds to various stages, as outlined in the study: Section 4.2.1 details the synchronization and size adjustment of the data conversion process. Section 4.2.2 discusses the methodology used to select the optimal combination of variables for the study. Section 4.2.3 explores how the best prediction lag time is determined in order to improve model accuracy. Section 4.2.4 presents the equation used in the multiple linear regression model. Section 4.2.5 outlines the settings for the ARIMA model. Finally, Section 4.2.6 details the parameter settings used in the LSTM model, providing insights into how each model is customized and optimized for a study’s specific requirements.

#### 4.2.1. Data Conversion

The climate data utilized in this study were sourced from the Environmental Information Open Platform provided by the Taiwan Ministry of Environment. This dataset comprised hourly observations from various regional meteorological stations. To ensure compatibility and consistency with the temporal resolution of other input variables in the predictive model, the climate data underwent conversion processes. Detailed instructions and methodologies for these data transformations are systematically presented in Table 5.

#### 4.2.2. Variable Selection

Selecting the right variables is a crucial step in any predictive modeling task. It involves identifying the most relevant features from a pool of potential variables in order to construct an effective model. Including irrelevant or redundant variables can lead to overfitting, where the model performs well on training data but poorly on unseen data. Multicollinearity, the phenomenon where two or more predictor variables are closely correlated, can further complicate the model’s ability to distinguish between their individual impacts. By carefully selecting variables, it is possible to mitigate redundancy, reduce multicollinearity, and enhance the stability of a model.

In the context of regression analysis, the adjusted R-squared value is a vital metric that refines the standard R-squared value to account for the number of predictors used in a model. It penalizes the model for including unnecessary variables, offering a more accurate measure of fit. This makes adjusted R-squared an ideal criterion for evaluating the effectiveness of different variable combinations, facilitating the comparison of models with varying numbers of predictors. Adjusted R-squared does not offer a clear explanation, and so it may be challenging for non-statisticians to understand its meaning. In addition, it can only be used for linear regression models and cannot be used for other types of models (such as nonlinear or machine learning models). Consequently, this study employs adjusted R-squared as the primary metric for variable selection.

**Figure 6:**
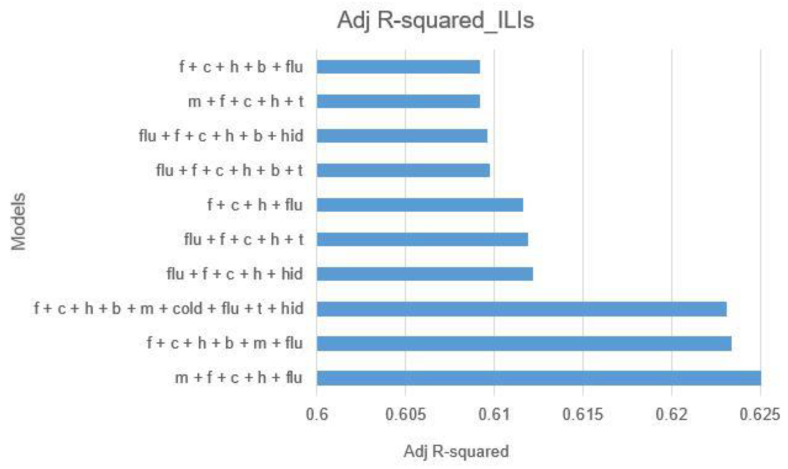
Adjusted R-squared with the output variable of ILIs identified the top 10 combinations. temperature (t), and humidity (h), with keywords encompassing fever (f), cough (c), muscle soreness (m), headache (h), fatigue (b), and influenza (flu).

This research utilizes three types of data: GT keywords, temperature (T), and humidity (H). The keywords used encompass fever (f), cough (c), muscle soreness (m), headache (h), fatigue (b), and influenza (flu). Temperature and humidity are denoted as t and hid, respectively. From these data, 120 different combinations of variables are possible. The evaluation performed using adjusted R-squared with the output variable of ILIs identified the top 10 combinations, as depicted in Figure 6.

The findings, illustrated in Figure 6, reveal that the combination of muscle soreness (m), fever (f), headache (h), and influenza (flu) achieves the highest explanatory power, with an R-squared of 0.62525. The next most effective combination includes fever (f), cough (c), headache (h), fatigue (b), muscle soreness (m), and influenza (flu), with an R-squared of 0.62342. A close third involves all variables, including fever, cough, muscle aches, headache, fatigue, cold, flu, temperature, and humidity, showing an R-squared of 0.62312. Although the combination of muscle soreness, fever, headache, and influenza exhibits the highest explanatory power, the marginal differences in R-squared values among the top combinations suggest minimal distinctions in their explanatory capabilities. Therefore, this study opts to utilize all variables for a comprehensive analysis, aiming to capture the broader dynamics of the model for use in subsequent predictions and analyses.

#### 4.2.3. Incubation Period and Time Lag

**Figure 7:**
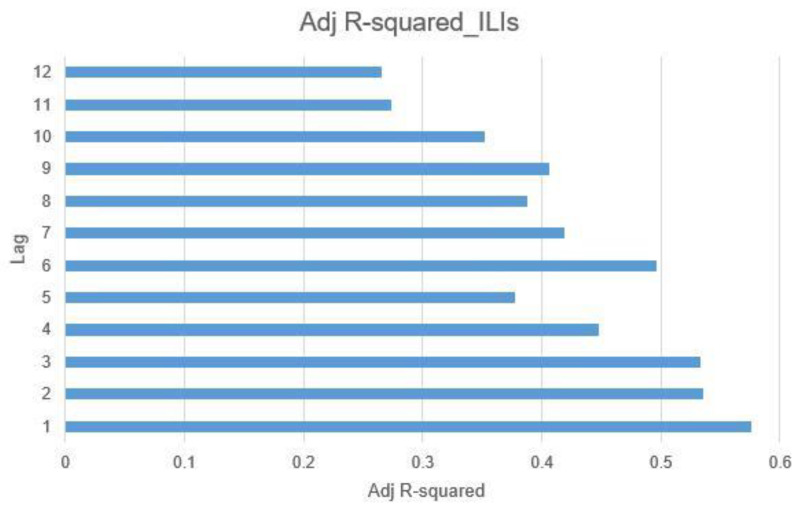
R-squared after Lag1~Lag12 adjustment.

The significance of the incubation period in the study and control of infectious diseases has been well-documented in previous research [87]. Echoing this, Chae et al. [84] and Kwon et al. [88] highlighted the predictive benefits of incorporating lag time into data analysis for the early detection of infectious diseases. Building on this insight, our study incorporated a lag time ranging from 1 to 12 weeks to observe its impact on predictive outcomes. An evaluation using adjusted R-squared with the output variable of influenza-like illnesses (ILIs) was conducted to assess the effectiveness of various lag times. The results, depicted in Figure 7, reveal that a 1-week lag time offers the highest explanatory power, with an R-squared value of 0.576089. Consequently, this study opts for a 1-week lag for further prediction and analysis, aligning with findings that the incubation period for ILIs is approximately one week long.

#### 4.2.4. Multiple Regression Analysis

The model’s establishment phase is entered after selecting the best variable combination in Figure 6 and selecting the best prediction time difference (Lag) in Figure 7. This study will use ${\hat{Y}}_{t}$ (dependent variable) as the number of influenza-like emergency cases, x (independent variable) to represent GT keyword indicators, and temperature and humidity as input items. The model is constructed as shown in Equation (9):

(9) $${\hat{Y}}_{t}=\beta_{1}GT_{i,t-1}+\beta_{2}T_{t-1}+\beta_{3}H_{t-1}+\varepsilon_{i}$$

where $\beta_{1}$ is the coefficient defining the influence on GT; $\beta_{2}$ is the coefficient defining the influence on T (temperature); $\beta_{3}$ is the coefficient defining the influence on H (humidity); and ε is the error. Furthermore, it is necessary to set the lag time difference to 1 in order to establish the regression equation.

#### 4.2.5. ARIMA Parameter Setting

**Figure 8:**
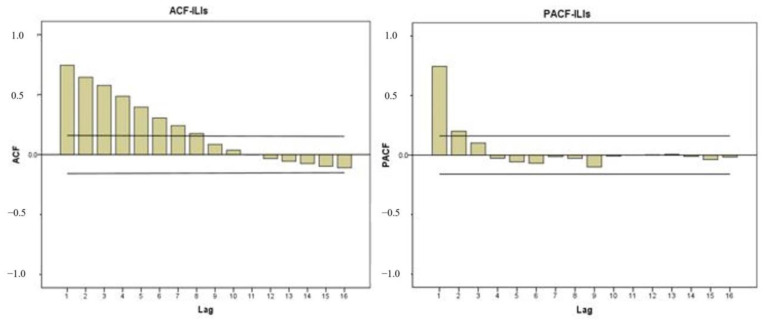
ACF and PACF inspection chart. Autocorrelation function (ACF) and partial autocorrelation function (PACF) tests.

ARIMA is among the most commonly used methods among statistical models for time-series prediction modeling. The model consists of three parameters (p, d, and q), and different parameter settings produce different results. The experimental parameter d used in this study is established based on the evaluation of whether the model data are stationary. After selecting the parameters p and q, the AIC (Akaike information criterion) and BIC (Bayesian information criterion) criteria are used to adjust p and q. Finally, the best model is selected for subsequent use. First, this study inputs GT keywords data, temperature, and humidity. It uses autocorrelation function (ACF) and partial autocorrelation function (PACF) tests to find out the truncation and tail of the model, as shown in Figure 8 below:

From the truncation and tail presented by ACF and PACF, we learn that the model enters the interval at AR (8). It enters the interval at PACF (2), and so the selection of ARIMA parameter values is based on the *p*-value set from 8 to 9. The d value is set from 0 to 1, and the q value is set from 2 to 3. The experiment tests various parameter combinations and finds the minimum RMSE value as the model input parameter result.

#### 4.2.6. LSTM Parameter Setting

Multiple regression models and time-series analysis ARIMA are traditional and standard analytical methods, and the LSTM method of deep learning has been proven to be suitable for the prediction of time-series data [65,78]. Therefore, this study uses LSTM by comparing its prediction performance with the ability of other two methods.

**Figure 9:**
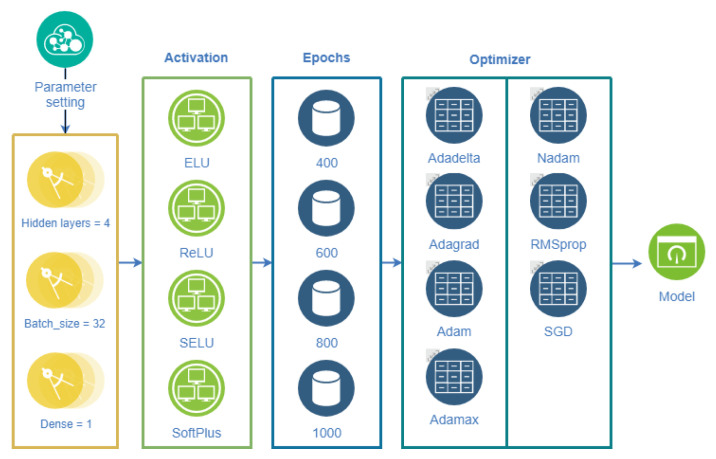
LSTM optimization parameter setting process.

To compare the pros and cons of the model, LSTM deep learning uses 23 parameters. Chae et al. (2018) [84] mention four activation functions (activation), for which ELU, ReLU, SELU, and SoftPlus are chosen, and four training periods (epochs), namely, 400, 600, 800, 1000, and 1200. Seven optimizers are used, which are Adadelta, Adagrad, Adam, Adamax, Nadam, RMSprop, and SGD. Various optimization parameter combinations are tested, with hidden layers set to 4, Batch_size is set to 32 (only 32 samples are trained each time), and dense is set to 1, resulting in 112 combinations, which are shown in Figure 9 to show the process of LSTM optimization of parameter settings. The minimum RMSE value is obtained as the output parameter of the model from the combination.

### 4.3. Evaluation Metrics

In order to assess the performance of the prediction models developed using three analytical learning approaches, this study employed two key metrics: mean absolute error (MAE) and root-mean-square error (RMSE). These metrics are pivotal in evaluating the accuracy of a model’s predictions, with lower values indicating superior predictive performances. The formulas used to calculate MAE and RMSE are presented as Equations (10) and (11), respectively.

(10) $$MAE=\frac{\sum_{n}^{i=1\;}\left|Y_{i}-\hat{Y_{i}}\right|}{n}$$

(11) $$R\mathrm{MSE}=\sqrt{\frac{1}{m}\sum_{i=1}^{m}{\left(y_{i}-{\hat{y}}_{i}\right)}^{2}}$$

## 5. Results and Discussion

The findings from this research are systematically presented in Section 5, with each subsection dedicated to a specific aspect of the study’s outcomes. Section 5.1 delves into the regression outcomes obtained for various combinations of variables using multiple linear regression. Section 5.2 discusses the forecasting results obtained from the ARIMA model. Section 5.3 elaborates on the predictions made by the LSTM model. Section 5.4 offers a comparative analysis and discussion of the models that demonstrated the greatest explanatory power. Finally, Section 5.5 explores the implications of the study infodemiology of ILI in clinical medicine.

### 5.1. Results of Multiple Linear Regression Model

In this study, the regression model incorporates a 1-week lag for each input variable, with the dataset split into training and testing segments at a ratio of 2:1. The experimental outcomes of the multiple linear regression model, across various variable combinations, are detailed in Table 6. According to the findings shown in Table 6, the variable combination of GT and temperature (T) exhibits the highest explanatory power, with an adjusted $R^{2}$ of 0.635. Following closely, the model that includes all variables (GT + temperature + humidity) ranks second in explanatory power, with an adjusted $R^{2}$ of 0.62727.

In order to delve deeper into the impact and significance of each variable within the regression model, Table 7 provides a comprehensive breakdown of the multiple linear regression outcomes for all variables. The results indicate that the *p*-values for the regression model concerning influenza-like illnesses (ILIs) are below the significance threshold (*p* < 0.05), suggesting that the model’s performance is statistically significant, with an adjusted $R^{2}$ value exceeding 0.5. This underscores the model’s substantial explanatory power, making it a reliable reference point. Notably, the search frequencies for the keywords ‘fever’ (*p* < 0.001) and ‘cough’ (*p* < 0.05) on Google Trends significantly influence the regression model. This implies that an increase in searches for these keywords to a certain threshold should prompt heightened vigilance from the Ministry of Health and Welfare. In addition, among climate factors, temperature (*p* < 0.05) has a more significant impact on ILIs than humidity.

### 5.2. Results of ARIMA Model

For comparative analysis, the ARIMA model’s input variables were aligned with those used in the multiple linear regression, with a one-week time lag and the division of the dataset into training and testing sets at 2/3 and 1/3 ratios, respectively. When configuring the ARIMA model’s parameters, autocorrelation function (ACF) and partial autocorrelation function (PACF) analyses were employed to determine the model’s cut-off and tailing effects. The model parameters were varied to explore a variety of parameter combinations, with the ‘*p*’ value set between 8 and 9, the ‘d’ value ranging from 0 to 1, and the ‘q’ value adjusted to between 2 and 3. As detailed in Table 8, this process resulted in eight distinct parameter configurations being evaluated.

Among these, the ARIMA (8, 0, 3) configuration demonstrated the most favorable results, achieving the lowest root-mean-square error (RMSE) value of 2550.76, alongside a robust explanatory power with an $R^{2}$ value of 0.796 and a standardized Bayesian information criterion (BIC) of 17.284. Consequently, the ARIMA (8, 0, 3) model was selected for use in the analysis of input variable combinations. The outcomes of applying the ARIMA (8, 0, 3) model, as shown in Table 9, reveal that the combined use of all variables (GT + temperature + humidity) in the model yielded the highest explanatory power, with an $R^{2}$ of 0.7966.

### 5.3. Results of LSTM Model

The LSTM model utilized in this study employs the same input variables as those used in the multiple linear regression and ARIMA models, with a time lag of 1 week. To determine the optimal parameter configuration for the LSTM model, the study explores various combinations of 7 optimizers, 4 activation functions, and 4 epoch settings, resulting in a total of 112 unique combinations. Table 10 outlines the enumeration of each optimization parameter, while Table 11 ranks the top-performing LSTM models based on their performance metrics.

According to the results presented in Table 11, the LSTM model configuration labeled as (5, 1, 4) emerges as the most effective, showcasing the lowest root-mean-square error (RMSE) value of 2923.31 and the lowest mean absolute error (MAE) value of 1588.55. Consequently, the study adopts the LSTM (5, 1, 4) configuration in order to analyze the input variable combinations in the subsequent phases.

The effectiveness of the LSTM (5, 1, 4) model’s application to various combinations of variables is detailed in Table 12. This analysis reveals that using the GT variable alone resulted in the lowest RMSE value of 2888.51, while the combination of GT and temperature (T) variables achieved the lowest MAE value of 1542.44.

### 5.4. Discussion and Comparison of Results across All Models

Table 13 compiles the evaluation results of all the models and their respective variable combinations, with the best evaluation indices highlighted in bold. Among the models, the multiple linear regression analysis, combining all variables (GT + T + H), achieves the most favorable result across all prediction models, with an RMSE value of 2413.33. Meanwhile, the ARIMA (8, 0, 3) model, utilizing only the GT variable, secures the best MAE value of 1218.98 among all the models evaluated. This underscores the ARIMA model’s proficiency in univariate time-series forecasting, and it is particularly crucial when capturing time-dependent patterns.

**Figure 10:**
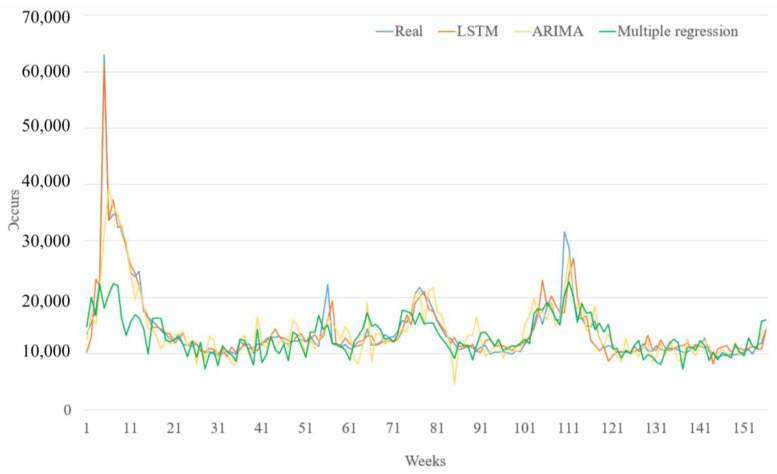
ILIs prediction results with all variables (GT + T + H).

Figure 10 juxtaposes the forecasted outcomes against the actual data utilized to predict ILIs by using the combined-variables model (GT + T + H). In Figure 10, we can observe that, in terms of the steady count of emergency room visits, the three models are almost comparable, but that in the early stages of the outbreak, the LSTM model performed well in the burst. This comparison reveals that, in scenarios of abrupt increases in the count of emergency room visits for influenza-like symptoms, the LSTM approach outperforms the other two methodologies, demonstrating its effectiveness in handling sudden shifts in the data trend.

The analysis encapsulated in Table 13 clearly demonstrates that the multiple linear regression model, when considering all variable combinations, outperformed both the ARIMA and LSTM models in predicting the volume of ILI emergency department visits. This suggests that regression analysis is a particularly effective tool for forecasting ILI-related emergency visits.

Further examination of the performance across different variable combinations revealed that the GT + H (Google Trends + humidity) combination yielded suboptimal predictions. In contrast, incorporating temperature (GT + T) significantly enhanced the model’s predictive accuracy. This indicates that temperature fluctuations, particularly during the influenza season, play a more crucial role in influencing ILI occurrences compared to humidity levels, highlighting the importance of temperature as a predictive factor for ILI emergency visits.

Statistically, the performance of the LSTM method is slightly lower than that of the regression and ARIMA methods. However, during the 2016 influenza outbreak, the LSTM method had a good prediction effect when there was an outbreak of influenza-like emergency rooms, but the performance could have been better. The possible reasons for the numerical value achieved are as follows:

1. Optimization of parameter selection: The choice of optimization parameters followed the approach of Chae et al. [78], with other parameters set to the default values seen in Keras. The optimal number of epochs remains uncertain due to the unique characteristics of each dataset, where data variability and stationarity can impact the ideal settings. The study’s fixed approach to hidden layers and batch sizes, without considering variable data factors, may have contributed to the reduced efficacy of the LSTM model.
2. Variable selection: At the optimal variable combination phase, despite identifying the top combinations, the study proceeded by using all variables for the LSTM model input. While LSTM models excel in analyzing complex time-series data with long-term dependencies, they require substantial data and computational resources. The exclusion of other potentially influential variables might have led to the LSTM model’s suboptimal learning outcomes.

This study faces several key limitations that warrant consideration. Firstly, the temporal scope of the data sources utilized for the model is relatively brief, spanning from 2016 to 2018. Notably, the year 2016 represented the peak of Google Trends (GT) activity, yet instances of influenza-like illnesses and confirmed influenza cases predate this period. Secondly, the dataset employed in this research aggregates the number of visits to emergency rooms for influenza-like symptoms on a weekly basis. Consequently, both GT and climate data were also consolidated into weekly intervals. This aggregation may not align optimally with the capabilities of the deep learning long short-term memory (LSTM) analysis method. The limited dataset size, particularly in the context of deep learning, which typically requires large volumes of data to achieve accurate predictions, could potentially diminish the effectiveness of the LSTM model’s predictive performance.

These insights suggest areas for further refinement in terms of model selection and parameter optimization in order to enhance predictive performance in future studies.

### 5.5. Implications of the Study Infodemiology of ILIs in Clinical Medicine

The study of infodemiology, particularly as it pertains to influenza-like illnesses (ILIs), carries significant practical implications for the field of clinical medicine. These implications span early detection, patient care, public health response, and health communication strategies. Outlined here are some of the implications of this study’s findings.

1. The discovery that Google Trends keywords for fever and cough significantly correlate with ILI incidence has practical uses, such as refining diagnostic criteria, enhancing public health surveillance, guiding health communication strategies, and informing clinical decision making and patient management during ILI outbreaks.
2. The practical implications of discovering that the incubation period of ILI is approximately one week in length include more accurate timing for public health advisories, targeted patient advice on symptom monitoring and quarantine durations, optimized timing for testing post-exposure, and improved models for outbreak prediction and management.
3. The finding that temperature is a more significant factor than humidity in monitoring the incidence of influenza-like illnesses implies that public health strategies and predictive models should prioritize temperature data in order to achieve more accurate surveillance, outbreak prediction, and intervention planning.
4. The finding that the ARIMA model has better explanatory power in terms of predicting influenza-like illnesses suggests its utility in enhancing disease surveillance systems, improving accuracy in forecasting outbreaks, and informing timely public health interventions and resource allocation decisions.

The practical implications of studying the infodemiology of ILI are profound and multifaceted, impacting public health surveillance, healthcare delivery, policymaking, and community awareness in several ways:

1. Early detection and preparedness: Infodemiology can alert clinicians and healthcare facilities to increases in ILI-related searches in their region, serving as an early warning system for potential outbreaks. This can prepare them for a surge in patient volume, enabling them to allocate resources effectively, such as staffing, beds, and medications.
2. Enhanced patient care: By understanding the prevalent symptoms and concerns of the population through search trends, clinicians can become more attuned to the needs of their patients. This insight can inform clinical assessments, diagnostic decisions, and patient communication, ensuring that care is responsive to the current epidemiological context.
3. Improved patient triage and management: Real-time data on ILI trends can help healthcare providers to triage patients more effectively, allowing them to prioritize care for those most in need and advise others on home care and symptom monitoring. This can also help in managing patient flow and reducing unnecessary hospital visits.
4. Telemedicine and remote care: Infodemiology’s insights can guide the implementation of telehealth services, identifying periods when remote consultations can effectively address patient needs and thus minimizing the risk of exposure for both patients and healthcare workers during flu seasons.
5. Vaccine promotion and administration: Clinicians can use infodemiology data to identify when and where to focus vaccine education and outreach efforts, particularly in areas showing high levels of ILI-related search activity but low vaccination rates.
6. Public health communication: Healthcare providers can leverage infodemiology findings to tailor their health communication strategies, addressing common misconceptions, providing clear guidance on when to seek medical care, and reinforcing preventive measures such as hand hygiene and vaccination.
7. Monitoring treatment efficacy and disease evolution: Infodemiology can aid in monitoring the effectiveness of treatment protocols and interventions over time, as well as in detecting shifts in disease presentation or severity, which may indicate emerging strains or changes in the ILI landscape.
8. Clinical research and epidemiological studies: Insights from infodemiology can inform clinical research priorities, helping to identify areas in need of further investigation, such as factors contributing to disease spread, the effectiveness of interventions, and public attitudes towards illness and prevention strategies.

In essence, the practical applications of ILI infodemiology in clinical medicine are vast, offering tools to enable better preparedness, patient care, and public health engagement. By integrating digital epidemiology insights with traditional clinical practices, healthcare providers can enhance their response to influenza-like illnesses, ultimately improving patient outcomes and public health.

Taiwan is one of the most advanced countries in the world in terms of providing health and medical data to people. The process of ILI surveillance, utilizing climate data, Google Trends, and ILI emergency case reports, can potentially be adapted and applied in other countries beyond Taiwan. The feasibility and effectiveness of such an integrated surveillance approach would depend on the availability, accessibility, and quality of the data in those countries. With the increasing digitization of health records and the prevalence of internet usage worldwide, many countries could leverage these data sources to enhance their public health surveillance systems, provided that there is a commitment to data sharing and the technical capacity for data analysis.

In addition, focusing on Google search data from specific professional communities, like medical doctors and nurses, for ILI surveillance could potentially increase the accuracy and relevance of predictions. These professionals might search for more technical terms related to ILI symptoms, treatments, and diagnostics compared to the general public, leading to the earlier and possibly more precise detection of outbreaks. However, this approach requires access to segmented data and may face privacy and data-sharing challenges. The best guess is that such targeted data could enhance predictive models but would need to be evaluated for its practical implementation and ethical considerations.

## 6. Conclusions

In today’s interconnected world, the ability to monitor outbreaks of influenza-like illnesses (ILIs) is vital for maintaining global health security. Early detection and swift responses are crucial in preventing the international spread of such viruses, thereby mitigating the risk of global pandemics. The surveillance of ILI trends yields critical data for researchers studying the virus, enhancing the understanding of its genetic diversity, transmission modes, and potential shifts in virulence. This knowledge is instrumental in refining vaccine formulations and developing more effective treatment strategies.

Surveillance efforts also assist health organizations in tracking the incidence and prevalence of ILI cases, which is crucial for monitoring disease spread and severity. Identifying patterns, hotspots, and vulnerable groups enables the implementation of timely interventions aimed at outbreak control.

Globally, ILI remains a significant public health concern. This study leverages data from Google Trends (GT), records of ILI-related emergency room visits, and climate information to forecast the ILI incidence in Taiwan. The findings indicate a correlation between GT data and ILI occurrences, underscoring the potential of GT to act as a supplementary tool for forecasting. According to the results from multiple regression analysis, variables such as fever, cough, and headache show a significant association with ILI cases (*p* < 0.05), highlighting the predictive value of GT data. Furthermore, the study reveals that combining GT with temperature data yields more accurate predictions than combining GT with humidity data, suggesting that temperature is a more reliable monitoring indicator.

Comparing different analytical approaches, regression analysis emerges as a particularly suitable method for this context. Although the deep learning LSTM model exhibits a slightly higher prediction error than traditional methods, its performance during the 2016 influenza outbreak demonstrates its capability to effectively predict ILI surges. Thus, despite its limitations, the LSTM method is recognized as a valuable tool in the arsenal of the analytical techniques used for ILI surveillance.

### Future Study

This study incorporates a limited range of external climate variables, specifically temperature and humidity, but excludes other potentially relevant climate data such as precipitation, sunshine hours, and wind speed. Including these additional variables could enhance the predictive accuracy of the influenza-like illness model. Furthermore, consistent with prior research, this study reaffirms the effectiveness of using variables from Google Trends (GT) as predictors of influenza outbreaks. When seeking future enhancements in the model’s performance, especially concerning the LSTM model, there is an opportunity to expand the range of optimization parameters and explore a broader array of model predictions. Adjusting the model’s learning rate and exploring diverse parameter combinations may address the current limitations related to model efficiency.

Additionally, while this study utilizes GT variables to represent the symptoms defined by the CDC, future investigations could benefit from examining a wider variety of GT variable combinations. This approach could further refine and improve the accuracy of model predictions for ILI, offering a more comprehensive understanding and forecasting capability.

## Figures and Tables

**Table 1:** Related work of influenza epidemics.

Disease	Data and Metrics	Method	Author
Flu or ILI	Climate	ARMA (autoregressive moving average model)	Huang [47]
		Mathematical modeling	Ayesha [48]
	Search engine data + traditional monitoring data	Pearson correlation coefficient	Ginsberg et al. [18]
			Cho et al. [49]
		Logit linear regression model	Kang et al. [50]
		ARGO autoregressive model	Yang et al. [2]
		HFSTM (Hidden Flu State from Tweet Model)	Prakash [51]
		Multiple regression model, artificial neural network	Xue et al. [52]
	Climate data + Google Trends	SARIMA (seasonal ARIMA), regression tree analysis	Zhang et al. [43]
		GLM (generalized linear model)	Dugas et al. [53]
		Negative binomial	Wisnieski et al. [54]
	Climate data + Google Trends + ILI emergency cases	Multiple regression model ARIMA, LSTM	Our study

**Table 2:** Related work on LSTM.

Method	Field	Indicator	Author
LSTM	Flow prediction	Traffic forecast	Tian et al. [71]; Yang et al. [72]; Zhao et al. [73]; Huang et al. [74]
		Stock forecast	Kim and Won [75]; Cao et al. [76]; Sonkavde et al. [77]
	Climate forecast	Temperature, pressure, humidity, dew point	Salman [78]; Narang et al. [79]
		Solar irradiance	Qing and Niu [80]; Jailani et al. [81]
	Health monitoring	Twitter, ILIs	Volkova et al. [82]; Akande et al. [83]
		Infectious diseases	Chae et al. [84]; Wan et al. [85]
		Sports performance	Wang et al. [86]

**Table 3:** Data source and variable description.

Variable	Source	Description	Number of Observations
Emergency influenza-like cases	Center for Disease Control	Weekly number of flu-like emergencies	156 weeks
GT keywords	Google Data Lab	Weekly Google search frequency	
Temperature	Environmental Protection Agency of the Executive Yuan	Average weekly temperature in Taiwan	
Humidity		Average weekly humidity in Taiwan	

**Table 4:** Descriptive statistics of variables.

Symptom	Variable	Min.	Median	Mean	Max.	Var.	SD
Number of ILI cases	Occurrences	9402	11,721	14,056.01	62,959	41,583,115.04	6469.27
Fever	GT frequency	44	67	67.36	100	103.68	10.22
Cough		34	57	60.21	100	196.09	14.05
Muscle soreness		6	40.5	41.05	100	287	17
Headache		54	76	75.06	100	59.37	7.73
Fatigue		11	44	44.29	100	256.74	16.07
Common cold		30	55.5	56.39	100	217.25	14.79
Influenza		1	11	14.73	100	200.67	14.21
Climate variables	Temperature (°C)	11.76	22.84	22.24	28.47	19.39	4.42
	Humidity (%)	65.97	78.78	78.14	86.76	16.7	4.10

**Table 5:** Data conversion.

Observation Data	Before Conversion	After Conversion Number of Input Samples
Station	36	36
Monitoring date	2016~2018	2016~2018
Monitoring time	Daily, hourly	156 weeks
Temperature		
Relative humidity		

**Table 6:** Regression results of various variable combinations.

Disease	Variable	$R^{2}$	$\mathrm{Adjusted}\;R^{2}$	F
ILIs	GT	0.663	0.609	12.355
	GT + T	0.692	0.635	12.078
	GT + H	0.663	0.601	10.592
	GT + T + H	0.693	0.627	10.537

Ps. T: temperature; H: umidity.

**Table 7:** Regression model results.

Disease	$R^{2}$	$\mathrm{Adjusted}\;R^{2}$	F	p	Variable	β	T	*p*
ILIs	0.693	0.627	10.537	<0.001	fever	255.983	4.054	0.000 ***
					cough	169.347	2.556	0.014 *
					muscle soreness	−57.750	−1.855	0.071
					headache	−97.753	−0.859	0.395
					fatigue	12.950	0.525	0.603
					common cold	−80.621	−0.948	0.349
					influenza	−61.738	−1.318	0.195
					temperature	−493.204	−2.015	0.049 *
					humidity	−49.845	−0.373	0.711

where *** *p* < 0.001, * *p* < 0.05.

**Table 8:** Results of ARIMA model.

Disease	Models	BIC	RMSE	MAE	$R^{2}$
ILIs	ARIMA (8, 0, 2)	17.223	2570.06	1240.5	0.786
	ARIMA (8, 1, 2)	17.545	2985.01	1352.04	0.718
	ARIMA (8, 0, 3)	17.284	2550.76	1270.35	**0.796**
	ARIMA (8, 1, 3)	17.656	3036.78	1517.76	0.718
	ARIMA (9, 0, 2)	17.330	2610.45	1241.22	0.786
	ARIMA (9, 1, 2)	17.674	3064.01	1475.43	0.713
	ARIMA (9, 0, 3)	17.419	2626.97	1278.88	0.790
	ARIMA (9, 1, 3)	17.715	3008.67	1351.63	0.732

The bold numbers represent the models with the best evaluation metrics.

**Table 9:** ARIMA model variable combination results.

Disease	Models	Variable	BIC	RMSE	MAE	$R^{2}$
ILIs	ARIMA (8, 0, 3)	Original	16.871	2921.32	1412.20	0.654
		GT	17.107	2519.19	**1218.98**	0.788
		GT + T	17.179	**2514.28**	1262.89	0.795
		GT + H	17.211	2554.76	1221.53	0.788
		GT + T + H	17.284	2550.76	1270.35	**0.796**

The bold numbers represent the models with the best evaluation metrics.

**Table 10:** Optimization parameter representative number.

Number	1	2	3	4	5	6	7
Optimizers	Adadelta	Adagrad	Adam	Adamax	Nadam	RMSProp	SGD
Activation	ELU	ReLU	SELU	SoftPlus	-	-	-
Epochs	400	600	800	1000	-	-	-

**Table 11:** Top ranked LSTM models.

Disease	Models	RMSE	MAE
ILIs	LSTM (5, 1, 4)	2923.31	1588.55
	LSTM (5, 3, 4)	2926.44	1589.58
	LSTM (5, 2, 4)	2930.74	1598.16
	LSTM (5, 3, 3)	3012.26	1579.25
	LSTM (1, 3, 3)	3018.78	1611.51
	LSTM (5, 1, 3)	3019.86	1588.94
	LSTM (1, 1, 3)	3035.9	1632.1
	LSTM (5, 2, 3)	3049.64	1623.26
	LSTM (1, 3, 4)	3060.79	1694.99
	LSTM (1, 4, 1)	3067.12	1806.03

**Table 12:** Variables combination results (LSTM).

Disease	Models	Variable	RMSE	MAE
ILIs	LSTM (5, 1, 4)	Original	2964.90	1589.26
		GT	**2888.51**	1663.13
		GT + T	2893.05	**1542.44**
		GT + H	3082.18	1768.11
		GT + T + H	2923.29	1588.07

The bold numbers represent the models with the best evaluation metrics.

**Table 13:** Comparison of explanatory power in all models.

Disease	Models	Variable	MAE	RMSE	$R^{2}$
ILIs	Regression analysis	GT	1705.3	2529.43	0.663
		GT + T	1624.18	2417.32	0.692
		GT + H	1699.5	2527.3	0.663
		GT + T + H	1623.67	**2413.33**	0.693
	ARIMA (8, 0, 3)	GT	**1218.98**	2519.19	0.788
		GT + T	1262.89	2514.28	0.795
		GT + H	1221.53	2554.76	0.788
		GT + T + H	1270.35	2550.76	**0.796**
	LSTM (5, 1, 4)	GT	1663.13	2888.51	-
		GT + T	1542.44	2893.05	-
		GT + H	1768.11	3082.18	-
		GT + T + H	1588.07	2923.29	-

The bold numbers represent the models with the best evaluation metrics.

## Data Availability

No new data were created or analyzed in this study. Data sharing is not applicable to this article.
